# Construction of a Mathematical Model of the Irregular Plantar and Complex Morphology of Mallard Foot and the Bionic Design of a High-Traction Wheel Grouser

**DOI:** 10.3390/biomimetics10060390

**Published:** 2025-06-11

**Authors:** Jinrui Hu, Dianlei Han, Changwei Li, Hairui Liu, Lizhi Ren, Hao Pang

**Affiliations:** 1 School of Agricultural Engineering, Jiangsu University, Zhenjiang 212013, China; 2222316034@stmail.ujs.edu.cn (J.H.); cwli@ujs.edu.cn (C.L.); 2212216053@stmail.ujs.edu.cn (H.L.); 2212316050@stmail.ujs.edu.cn (L.R.); 2 Key Laboratory of Modern Agricultural Equipment and Technology, Ministry of Education, Jiangsu University, Zhenjiang 212013, China; 3 School of Mechanical and Aerospace Engineering, Jilin University, Changchun 130022, China; panghao@jlu.edu.cn

**Keywords:** bionic design, plantar morphology of mallard foot, mathematical model construction, high-traction wheel grouser, sand fixation and flow restriction characteristics

## Abstract

To improve the traction performance of mobile mechanisms on soft ground, such as paddy fields, tidal flats, and swamps, a mallard (Anas platyrhynchos) foot was adopted as a bionic prototype to explore the influence and contribution of the plantar morphology of the toes and webbing on the anti-subsidence function during its locomotion on wet and soft substrates and to apply this to the bionic design of high-traction wheel grousers. A handheld three-dimensional laser scanner was used to scan the main locomotion postures of a mallard foot during ground contact, and the Geomagic Studio software was utilized to repair the scanned model. As a result, the main three-dimensional geometric models of a mallard foot during the process of touching the ground were obtained. The plantar morphology of a mallard foot was divided into three typical parts: the plantar irregular edge curve, the lateral webbing surface, and the medial webbing surface. The main morphological feature curves/surfaces were extracted through computer-aided design software for the fitting and construction of a mathematical model to obtain the fitting equations of the three typical parts, and the mathematical model construction of the plantar irregular morphology of the mallard foot was completed. In order to verify the sand-fixing and flow-limiting characteristics of this morphological feature, based on the discrete element method (DEM), the numerical simulation of the interaction between the plantar surface of the mallard foot and sand particles was carried out. The simulation results show that during the process of the mallard foot penetration into the loose medium, the lateral and medial webbing surfaces cause the particles under the foot to mainly move downward, effectively preventing the particles from spreading around and significantly enhancing the solidification effect of the particles under the sole. Based on the principle and technology of engineering bionics, the plantar morphology and movement attitude characteristics of the mallard were extracted, and the characteristics of concave middle and edge bulge were applied to the wheel grouser design of paddy field wheels. Two types of bionic wheel grousers with different curved surfaces were designed and compared with the traditional wheel grousers of the paddy field wheel. Through pressure-bearing simulation and experiments, the resistance of different wheel grousers during the process of penetrating into sand particles was compared, and the macro–micro behaviors of particle disturbance during the pressure-bearing process were analyzed. The results show that a bionic wheel grouser with unique curved surfaces can well encapsulate sand particles at the bottom of the wheel grouser, and it also has a greater penetration resistance, which plays a crucial role in improving the traction performance of the paddy field wheel and reducing the disturbance to the surrounding sand particles. This paper realizes the transformation from the biological model to the mathematical model of the plantar morphology of the mallard foot and applies it to the bionic design of the wheel grousers of the paddy field wheels, providing a new solution for improving the traction performance of mobile mechanisms on soft ground.

## 1. Introduction

Tidal flat areas are widely distributed, and coastal zones with abundant sediment resources are still expanding due to siltation. Thus, tidal flats exhibit advantages such as a large area, rapid siltation, renewability, and great potential. At the same time, tidal flats can be used as important reserve land resources, which can not only help to achieve the dynamic balance of total cultivated land but also promote agriculture [1,2], fisheries, forestry, animal husbandry, and salt industry development. However, tidal flats feature a soft and unstable substrate with deep, sticky silt, making conventional vehicle walking mechanisms difficult to operate. This hinders the development, utilization, and protection of tidal flats. Moreover, the key to achieving mechanized operations on tidal flats is to solve the passability issue of mobile mechanisms.

In addressing this mobility challenge, bionics can provide new ideas and directions. Bionics, as fast and low-cost ways to maximize scientific discovery and technological innovation, effectively solve various problems in the process of technology development and meet the various needs of human production and life [3]. Many animals in nature have their own unique plantar topography, which helps them better adapt to diverse environmental conditions. Camels living in desert areas have a strong adaptability to the desert. Camel feet are two-toed, the thick toe pads on the soles of the feet are elastic devices, and they can have a good vibration-slowing effect when moving on soft sandy ground and hard ground, and there is a smooth concave shape in the middle of the soles, which plays a role in sand fixation and current restriction [4]. Goats inhabit steep slopes and uneven terrain all year round, such as mountains, hills, and rocky areas. They move freely in complex mountain terrain due to their foot morphology: obliquely concave soles that fix the medium in grooves to restrict flow and hooves that open to grip rocks tightly during movement, preventing sole slippage [5]. The turtle (*Caretta caretta*) exhibits excellent locomotor performance on both hard and sandy surfaces, and the webbed turtle feet use their claws to improve stability during hard ground movements and do not slip when moving on sandy ground, mainly due to the formation of hardened areas behind their webbed turtle feet, which restricts the flow of plantar media [6]. The North American river otter has a series of soft plantar pads on its paws, with a textured epidermal layer surrounding them, which constitutes a unique grooved soft tissue that allows it to drain plantar fluid well and maintain grip when it touches the ground [7]. The tarsal nodes of the stonefly are cylindrical in shape, and its smooth and hairy attachment surface helps the stonefly move horizontally and vertically on a variety of media [8].

Applying the excellent locomotion mechanisms of living things over hundreds of millions of years of evolution to engineering design is what bionics is all about [9,10,11,12,13]. Chen et al. designed the “Sand Bot” to study the mechanics of the zebratail lizard’s legs, feet, and substrate interaction, as well as its high locomotion performance in the desert, to address the challenge of traversing soft sand surfaces [14]. In order to further apply biological features to engineering design, some scholars have transformed biological models into mathematical models through reverse reconstruction technology [15,16]. Zhang et al. obtained the 3D point cloud data of the irregular surface at the bottom of the goat’s hoof through reverse engineering technology and used the Matlab software fitting method to mathematically model the surface at the bottom of the goat’s hoof so as to realize the transformation of the irregular surface at the bottom of the goat’s hoof from biological model to mathematical model [17]. At the same time, based on the curve equation of the bottom of the goat’s hoof, Zhang et al. applied it to the design of the tire tread structure and designed a tire with a bionic pattern, which significantly improved the traction performance of the tire [18]. Zhou et al. designed a bionic tire surface inspired by the curved surface of the third toe of the ostrich foot, and the bionic wheel was experimentally verified to have good anti-sinking and sand fixation properties, as well as improved traction and bearing capacity [19]. Godon et al. developed a bionic foot with split hooves for a large quadruped robot, drawing inspiration from the moose’s hoof structure, enabling efficient movement on wet and soft terrain [20]. These studies are of great significance for overcoming the passability problem of mobile mechanisms on soft ground.

Of course, solving the problem of the passability of the moving mechanism on soft soil can not only change the wheel and wheel surface structure [21,22,23]; a certain height of wheel grousers can also be installed on the rim to improve the traction performance of the wheel [24,25]. It is clear that wheel grousers [26] play an important role in the friction of the wheels against the soft ground, and most of the early lunar rovers also added cones [27] or rectangular wheel grousers to the surface of the wheels [28]. Recently, a number of studies have been conducted on the wheel structure used on soft soils and the interaction between wheel and soil, but the wheel grouser is still the traditional rectangular wheel grouser [29,30,31], neglecting to innovate the wheel grouser structure. Yuan et al. designed a high-traction tracked wheel grouser based on the structural characteristics of ostrich feet, which significantly improves the traction performance of tracked wheels in wet and soft paddy fields [32]. Mallards inhabit tidal flats all year round and can move on soft ground such as river beaches and swamps without being prone to slippage and subsidence, and their special webbed foot structure has made a significant contribution. The webbed feet of mallards allow them to fix loose particles on the soles of the feet well when moving on soft ground, which plays a role in sand fixation and flow restriction. So far, no scholar has applied this characteristic to the structural design of wheel grousers.

**Figure 1:**
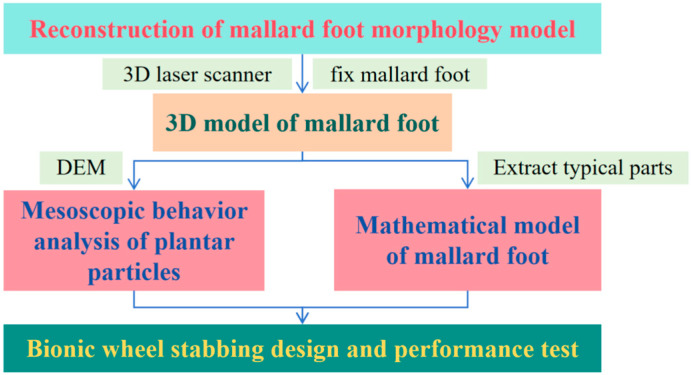
Design flowchart.

Therefore, this paper takes the mallard foot as a bionic prototype. After verifying the sand fixation and current limiting characteristics of the mallard plantar morphology, this study then applies its morphological characteristics to the structure of the bionic wheel grouser, designs two bionic wheel grousers, and compares them with the two traditional wheel grousers by discrete element method to verify their traction performance and sand fixation and current limiting characteristics, and analyzes the role of the bionic wheel grouser surface in improving traction. The overall design process is shown in Figure 1.

## 2. A 3D Model of the Plantar of Mallards

### 2.1. Reconstruction of a Mallard Foot Morphology Model

**Figure 2:**
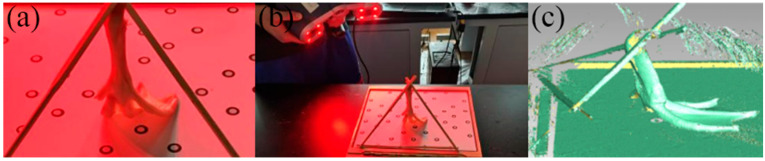
Scan of a 3D model of a mallard foot. (**a**) Fixed mallard foot; (**b**) scanning the mallard foot; and (**c**) the scan result.

In this study, the right foot of a healthy male mallard, reared under free-range conditions for two years in Jiaxing, Zhejiang Province, was selected as the research object. Using a handheld self-positioning 3D laser scanner (Creaform EXAscan, Lévis, QC, Canada) [33], we scanned the mallard’s feet. During mallard locomotion, the distal phalanges of the feet first touch the ground, the webbing between the toes is slightly closed, and then the whole palm touches the ground; the webbing between the toes is fully open, the touching area reaches the maximum, the hind palm leaves the ground first, the webbing between the toes is closed, and the mallard paw takes the tip of the toes as the fulcrum and rotates at an angle with the sand surface to leave the sand surface. This sequence of foot movements was simplified, and the toe posture during ground contact in the stance phase was selected for 3D scanning. Tools such as cardboard, glue, wire, and chopsticks are used to help fix the mallard foot, as shown in Figure 2a. A handheld self-positioning 3D laser scanner was used to scan the fixed mallard foot, as shown in Figure 2b, to obtain the 3D point cloud data of the mallard foot [34], as shown in Figure 2c.

**Figure 3:**
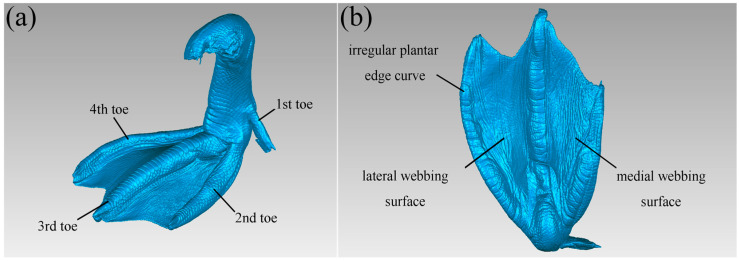
Morphology of toe-webbed structure of a mallard foot in the middle of ground touchdown. (**a**) Mid-touchdown top view; (**b**) mid-touchdown plantar features.

Since the laser scanner also captures surrounding objects, the initial scanned model contains a significant amount of noise and does not represent a complete mallard foot. Additionally, due to the small size and uneven surface of mallards, some areas are difficult to scan, so defects such as vulnerabilities can occur, and so the reverse engineering software Geomagic Studio 2014 (Geomagic Corporation, Cary, NC, USA) is required; a series of restoration works were carried out on the model to obtain the ideal mallard geometry. The scanned file was saved in the .stl format and imported into the reverse engineering software Geomagic Studio for denoising, feature removal, hole filling, and other processing to obtain a smooth mallard geometry, as shown in Figure 3.

### 2.2. Mathematical Model of Mallard Soles

It is not easy for mallard feet to slip and sink when moving on soft ground such as tidal flats and mud, which is closely related to the structure and morphology of their toes and webs. As previously described, during the mid-stance phase of movement, the entire foot makes contact with the ground, and the webbing between the toes is fully extended. At this point, the ground contact area reaches its maximum, making it the most critical phase in terms of ground interaction.

Observation of the toe-webbing structure of mallard feet in the middle of the ground (Figure 3) showed that a mallard foot has four toes, and the mallard foot was webbed between the second, third, and fourth toes, and the webbing was thin, the surface was textured, and the touch was rough and dry. Due to natural evolution and environmental adaptation, the first toe of the mallard foot has gradually degenerated and is positioned backward, making no contact with the ground during movement. Therefore, it can be excluded when extracting the toe-webbing structure and morphology, and its morphological characteristics will not be considered in the subsequent research. The mallard primarily relies on the second, third, and fourth toes for locomotion and body support. During ground contact, these toes increase the contact area, contributing to sand fixation, flow resistance, anti-slipping, and improved walking efficiency. The unique toe-webbing structure of the mallard enables its effective movement on soft terrain such as tidal flats. Therefore, we divided the soles of mallards into three typical parts: the plantar irregular edge curve, the lateral webbing surface, and the medial webbing surface to extract the toe-webbing structure and morphological characteristics of mallards. However, these three typical features are irregular and cannot be directly observed and measured, and these biological feature models need to be transformed into mathematical model analyses.

#### 2.2.1. Mathematical Model of Mallard Plantar Irregular Edge Curve

**Figure 4:**
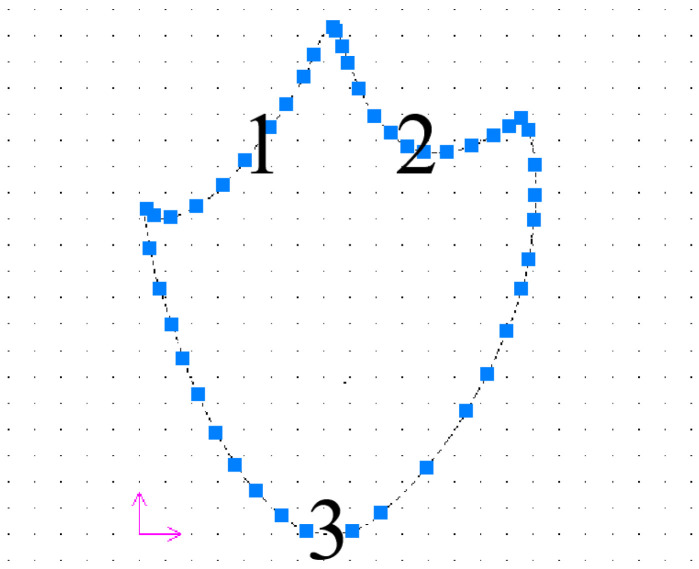
Curve of the edge of the mallard foot. In the figure, 1, 2, and 3 are three parabolas formed by dividing the mallard irregular plantar edge curve. The pink arrows indicate the directions of the two-dimensional coordinate axes, the horizontal arrows represent the X-axis direction, and the vertical arrows represent the Y-axis direction.

The scanned mallard model was first aligned to a standard 3D coordinate system using the Geomagic Studio software. Subsequently, the processed model was imported into SolidWorks 2021 (Dassault Systèmes Corporation, Concord, MA, USA). In SolidWorks 2021, the .stl file of the aligned ideal mallard foot model was opened, and the overall plan of the mallard foot was identified. By leveraging the spline function within the sketching module, the irregular edge curves of the mallard were meticulously mapped onto the edge profile of the original mallard foot. This process ensured that the edge curves were refined to achieve a smooth and complete representation, thereby enhancing the geometric accuracy and visual quality of the model. Finally, a new drawing was created in SolidWorks, and the 3D model of the mallard foot was imported to generate a corresponding 2D technical drawing. The CAXA (Beijing Digital Dafang Technology Co., LTD, Beijing, China) electronic drawing board was then used to open the 2D diagram and create a new suitable coordinate system, as shown in Figure 4.

The mallard irregular plantar edge curve was divided into three parabolas according to the shape, the coordinate points were evenly marked on the parabola, and the coordinate points were saved as .txt format files, and then the curves were fitted using the EXCEL (Microsoft, Redmond, WA, USA) software, and the fitting equations of the mallard plantar irregular edge curves were obtained, as shown in Equations (1)–(3).

(1) $$y=0.0162x^{2}-0.1493x+121.12\;2<x<72$$

(2) $$y=0.0246x^{2}-5.8117x+485.70\;72<x<146$$

(3) $$y=0.0223x^{2}-3.3019x+119.17\;2<x\leq 146$$

The coefficients of determination R^2^ of Equations (1), (2) and (3) are 0.9987, 0.9808, and 0.9435, respectively, all close to 1. This shows that the biological model has been successfully transformed into a mathematical model and that the equation fits to a very high degree.

#### 2.2.2. Mathematical Modeling of the Lateral and Medial Webbed Surfaces of Mallards

**Figure 5:**
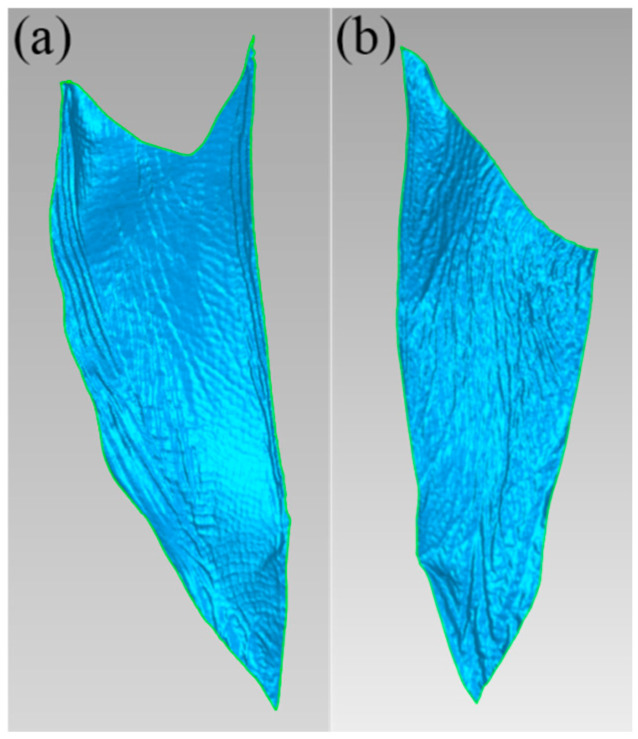
Curved surface of two fins of a mallard foot. (**a**) Lateral webbed surface; (**b**) medial webbed surface.

The lateral and medial flipper models of the mallard foot were obtained by cropping the mallard model in Geomagic Studio, as shown in Figure 5. The cut patches were converted to points and saved as .asc files, and then the surface fitting was performed using the MATLAB R2021a (MathWorks, MA, USA) software, and the fitting results are shown in Table 1 and Table 2.

Analysis of Table 1 and Table 2 shows that when fitting to *x*^2^*y*^2^, the values of the residual sum of squares (SSE) and mean square deviation (RMSE) are small, and the value of the coefficient of determination (R^2^) is large and close to 1, indicating that the accuracy of the equation is high. Therefore, the equations for which the exponents of *x* and *y* are selected as quadratic yields are obtained as shown in Equations (4) and (5), respectively.

(4) $$\begin{array}{c} z=25.41-0.2624x+1.603y-0.0029xy\\ -0.0046x^{2}+0.0281y^{2}+0.0001xy^{2}+0.0008x^{2}y \end{array}$$

(5) $$\begin{array}{c} z=7.844-0.1921x-0.2852y+0.0158xy\\ -0.0021x^{2}-0.0154y^{2}+0.0003xy^{2}-0.0003x^{2}y \end{array}$$

## 3. Mesoscopic Behavior Analysis of Mallard Plantar Particles

### 3.1. Discrete Element Simulation Intrusion Test

The mallard plantar surface consists of the lateral webbed surface, the medial webbed surface, and the second, third, and fourth toes, which together form a unique toe-webbing morphology. This morphological feature is characterized by a concave center and a raised edge, which plays a decisive role in the interaction between mallard feet and loose substrates. Therefore, studying the mesoscopic behavior of particles beneath the plantar surface has both theoretical and practical significance for constructing a mathematical model of the mallard’s plantar morphology.

**Figure 6:**
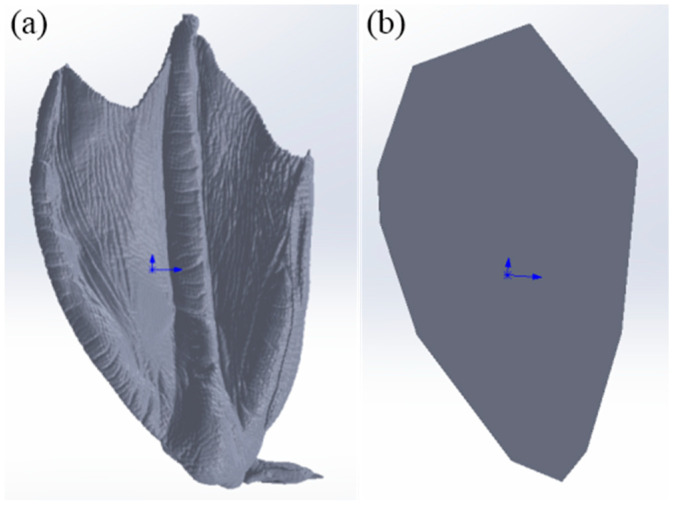
Two foot-end models. (**a**) Mallard foot model; (**b**) contrast foot model.

In the investigation of the role of the mallard’s unique plantar morphology in sand fixation, flow resistance, and anti-subsidence, simulation intrusion tests were conducted to accurately assess its impact on anti-subsidence performance. Using the Discrete Element Method, a mallard foot model and a contrast foot model (Figure 6) were developed for comparison. The contrast foot was designed based on the shape of the mallard plantar surface and had the same plantar area as the mallard foot model. After importing these two types of foot-ends into the EDEM 2022 (Discrete Element Method, Altair Corporation, Troy, MI, USA) software, a force of 12N was applied to them, and an intrusion operation was carried out on the sand particles in a square soil tank with dimensions of 0.25 × 0.25 × 0.15 m at an initial velocity of 0.005 m/s. The termination time set for this simulation experiment was 1.2 s. In the simulation test, the dry sand particles were spherical with a radius of 1 mm, had a density of 1482 kg/m^3^, the Poisson’s ratio was 0.3, and the shear modulus was 2.82 × 10^8^. The Hertz–Mindlin with JKR Cohesion (JKR) contact model was used for the particle model, where the recovery coefficient was 0.41, the static friction coefficient was 1.01, the rolling friction coefficient was 0.115, and the JKR surface energy was 0.024 J/m^2^ [35,36].

### 3.2. Behavior Analysis of Sand Particles

**Figure 7:**
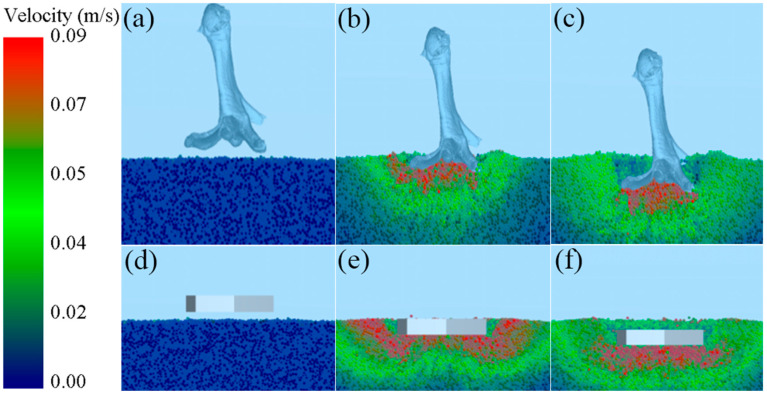
Dynamic velocity field of underfoot particles. (**a**) Commencement of intrusion; (**b**) mid-intrusion; (**c**) end of intrusion; (**d**) commencement of intrusion; (**e**) mid-intrusion; and (**f**) end of incursion.

**Figure 8:**
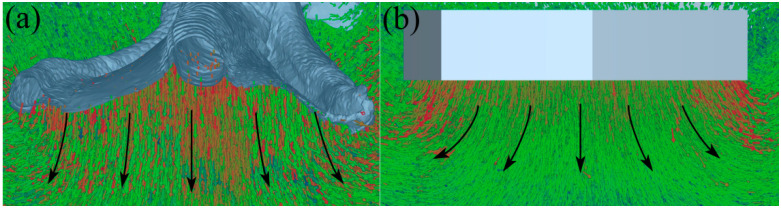
Direction of movement of the foot particles. (**a**) Mallard foot; (**b**) contrast foot. The red arrows represent the movement direction of the particles with faster speeds, the green ones represent those with slower speeds, and the black ones represent the overall movement direction of the particles.

As shown in Figure 7, the dynamic velocity field of sand particles in a simulated intrusion test is shown [37], where the velocity of the particles identified by warm colors is relatively large, while that of the particles identified by cool tones is relatively small. As shown in Figure 7a–c, particles with higher velocities are mainly distributed beneath the foot and along the edges of the toes during the mid-stage of mallard foot intrusion. At the end of the intrusion, particles with a higher velocity are mainly concentrated under the foot. It can be observed that as the intrusion progresses, the disturbance caused by the mallard foot is primarily focused beneath the foot, while disturbances to the edges of the toes and particles farther from the foot end are less significant. As can be seen from Figure 7e–f, the contrast foot disturbed most of the particles in the plantar and marginal areas of the foot during the middle stage of intrusion. At the end of the intrusion, most of the particles with higher velocity are concentrated in the underfoot and marginal areas of the foot. As shown in Figure 8a,b, due to the flattening of the plantar surface of the contrast foot, the particles beneath the foot primarily move outward as the contrast foot intrudes downward. Compared with the contrast foot, with the downward intrusion of the mallard foot, the morphological characteristics of the lateral webbed surface, medial webbed surface, and toe of the mallard foot formed by the middle concave and convex edge make the underfoot particles mainly produce a downward movement trend but are not easy to move in the surrounding direction.

During the intrusion process, the morphological characteristics of the middle concave and edge bulge of the mallard foot made the disturbance of the underfoot particles mainly become concentrated under the foot. This feature enables mallards to apply force more effectively on the underfoot area when moving on loose substrates, providing more stable support for the body, reducing displacement of the toe edges and distal particles, and lowering the risk of imbalance caused by substrate flow. Meanwhile, the structures of the lateral and medial webbed surfaces promote a downward movement trend of the particles when compressed, effectively inhibiting their lateral diffusion. This compacts the substrate, improves bearing capacity, and significantly reduces the depth of subsidence. This structural characteristic allows mallards to greatly improve their mobility efficiency on loose substrates, allowing them to walk and forage more efficiently in sandy areas, river beaches, and other environments.

## 4. Bionic Wheel Stabbing Design and Performance Test

### 4.1. Bionic Wheel Grouser Design

**Figure 9:**
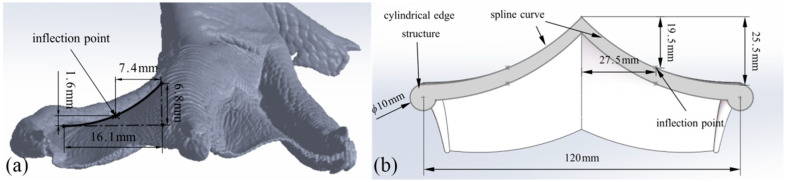
WBWG design. (**a**) Lateral webbed surface curve; (**b**) webbed bionic wheel grousers (concave).

Based on the unique morphological characteristics of a mallard foot with a concave middle and a raised edge, and based on the fitting equation of the lateral webbed surface (Equation (4)), the surface curve data (Figure 9a) of the mallard foot was extracted as a biomimetic prototype to design a webbed bionic wheel grouser (WBWG). As shown in Figure 9b, the WBWGs adopt a left–right symmetrical structure, and the spline curve tool in the SolidWorks software is used to accurately reproduce the prototype curve shape so as to ensure that the position relationship between the inflection point of the curve on the surface of the wheel grouser is highly consistent with the geometric characteristics of the lateral webbed surface curve of the mallard foot. In addition, a cylindrical edge structure is designed at the edges of both ends of the wheel grouser, which is similar to the mallard toe, aiming to optimize the grip performance and movement efficiency of the wheel grouser on soft ground through the bionic structure.

### 4.2. Discrete Element Simulation Experimental Design

**Figure 10:**
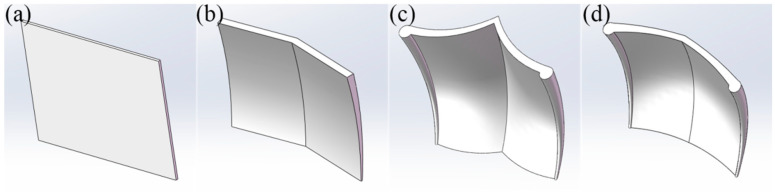
Four different wheel grouser models. (**a**) Flat wheel grouser; (**b**) herringbone wheel grouser; (**c**) WBWG-C; and (**d**) WBWG-V.

In order to verify the effectiveness of the webfoot bionic wheel grouser design, discrete element simulation experiments were carried out using the EDEM software to simulate the interaction process between four different wheel grousers (Figure 10) and soil [38]. The test samples include two types of anti-slip iron wheel grousers for traditional micro-tillers, namely a flat wheel grouser and a herringbone wheel grouser, as well as two types of bionic wheel grousers designed based on the foot morphology features of mallards, namely a webbed bionic wheel grouser (concave) (WBWG-C) and a webbed bionic wheel grouser (convex) (WBWG-V). The bottom surface area of each wheel grouser is controlled to be the same to ensure the singularity of the test variables.

In the experiment, sand particles were selected as the soil medium, and the particle parameters were the same as those in the discrete element simulation intrusion test. In the square soil trough with a size of 0.25 × 0.25 × 0.15 m, it was pre-filled with 2/3 of the volume of sand particles to ensure that the wheel grousers could be completely submerged. After particle generation was completed, the wheel grouser was placed on the particle surface. To simulate the actual driving process, in which the anti-skid iron wheel grouser pulls the soil backward, a rotational speed of 3 rad/s was applied. This setup helped eliminate interference from speed variation in the test results. The time interval of the wheel stabbing movement was set from 1.14 s to 1.72 s to capture the critical stage of its interaction with the soil.

After the simulation, the mesoscopic motion behavior of the particles in the process of wheel grouser soil penetration was observed through the post-processing function of the EDEM software, and the traction force provided by different wheel grousers was quantitatively compared and analyzed, and the effectiveness of the wheel grouser design was evaluated from the traction force and soil disturbance.

### 4.3. Comparative Analysis of Wheel Stabbing Performance

**Figure 11:**
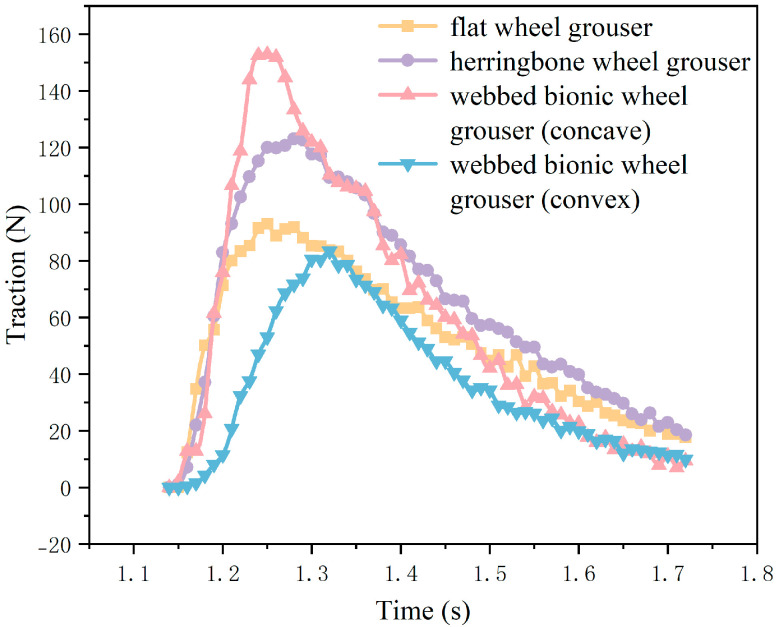
Comparison of traction performance.

As shown in Figure 11, the comparison of the traction of the WBWG with the same motion parameters as the traditional anti-skid iron wheel grouser is shown. It can be observed that the traction forces generated by the four wheel grousers increase rapidly after initial contact with the soil, then gradually decrease after reaching their respective peaks. The traction of the flat wheel grouser, herringbone wheel grouser, and WBWG-V all reach a peak at about 1.3 s and then gradually decline, and the overall ascent and descent processes are relatively smooth. Among the four types, the herringbone wheel grouser provides relatively higher traction. The ascent phase of the WBWG-C is particularly steep, reaching its peak at approximately 1.25 s. Moreover, this peak traction is significantly higher than that of the other three grousers, indicating that the WBWG-C can deliver superior traction performance within a very short period of time.

**Figure 12:**
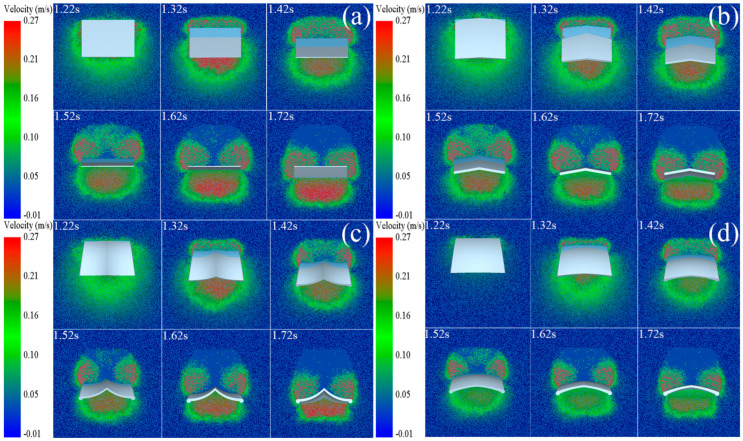
Comparison of soil disturbances. (**a**) Flat wheel grouser; (**b**) herringbone wheel grouser; (**c**) WBWG-C; and (**d**) WBWG-V. Warm tones represent particles with higher speeds, while cool tones represent particles with lower speeds.

Figure 12 shows the velocity contour of the WBWGs with the same motion parameters as the traditional non-skid iron wheel grousers disturbing the soil particles, where the warm colors represent the faster particles and the cool colors represent the slower particles. As shown in Figure 12a,b, it can be seen that the flat wheel grouser only squeezes and accumulates the soil particles backwards in the process of soil picking and cannot gather the soil particles at the bottom. In contrast, the structural design of the herringbone wheel grouser makes it provide a certain horizontal component to the bottom particles in the process of soil pulling, which then produces a certain convergence effect on the bottom particles, but the effect is not significant. As shown in Figure 12c,d, it can be seen that, compared with the traditional wheel grousers, the two WBWGs show good sand fixation and polysalience by virtue of their unique morphology and cylindrical edge design, among which the WBWG-C is particularly prominent. As can be seen from Figure 12c, at 1.72 s, compared with the other three types of wheel grousers, the particles with higher velocities basically gather only at the bottom of the WBWG-C. Additionally, this configuration causes minimal disturbance to particles in other regions at the bottom.

In summary, compared with traditional wheel grousers, WBWG-C can provide greater traction when they first touch the ground, preventing wheel sagging and providing better traction performance. When touching the soil, the soil is gathered at the bottom, which has less soil disturbance than other areas, avoids damage to other soil layers, and greatly improves the passing performance and anti-subsidence ability of agricultural equipment.

These excellent performances can be attributed to their unique webbed-foot-like structural design. The concave bionic wheel grousers (with a webbed foot morphology) are morphologically highly similar to the plantar structure of mallard feet during the mid-stance phase of ground contact. Both exhibit geometric features of a central depression and marginal elevation, along with an inwardly concave webbed surface structure. During soil contact, this special geometric configuration alters the direction of particle forces, inducing a significant vertical displacement trend in the particles beneath the sole. This vertical movement not only effectively compacts the sand particles in the contact area, significantly enhancing the structural bearing capacity, but also reduces the settlement depth of the structure by enhancing inter-particle interlocking and frictional forces. Additionally, the cylindrical edge structures of the bionic wheel grousers, which mimic the toes of the mallard foot, act synergistically with the inwardly concave webbed surface to further reduce lateral diffusion of sand particles by constraining their lateral displacement. The effectiveness of this bionic design indirectly confirms that the special plantar morphology exhibited by mallards during the mid-stance phase is highly likely an optimized locomotory adaptation strategy evolved through long-term exposure to soft substrate environments.

However, this study has certain limitations. Although we designed webbed-foot bionic wheel grousers through engineering bionics and verified their sand-fixing and flow-limiting performance, we did not conduct physical performance tests in field environments. Due to the coupled effects of factors such as soil mechanical properties, environmental temperature/humidity, and complex terrain in actual engineering applications, the applicability of the current research findings under real-world working conditions requires further verification. Moreover, there is still room for optimization in material selection, surface texture structure, curvature parameters, and anti-adhesion performance of the bionic wheel grouser. To address these shortcomings, future research could (1) fabricate bionic wheel grousers with different curvature parameters using various materials; (2) replicate the surface texture structure of mallard feet via reverse engineering to investigate its mechanism of action on soil adhesion/desorption behavior; and (3) integrate the optimized bionic wheel grouser into agricultural equipment (e.g., micro-tillers, rice transplanters) to conduct multi-condition, multi-environment field trials, systematically evaluating their locomotory performance, load-bearing capacity, and durability in complex agricultural scenarios to provide more comprehensive technical support for their engineering application.

## 5. Conclusions

In this study, a 3D geometric model of a mallard foot was obtained by scanning with a handheld 3D laser scanner, and an ideal geometric model of a mallard foot was obtained by a series of restoration works with the help of the reverse engineering software Geomagic Studio. After analyzing the characteristics and morphology of the plantar of the mallard foot, the plantar of the mallard foot was divided into three typical parts: the irregular plantar edge curve, the lateral webbed surface, and the medial webbed surface. The fitting equations of three typical parts were obtained by computer-aided design software (SolidWorks, Geomagic Studio, CAXA) and data analysis software (EXCEL and MATLAB), and a mathematical model of the irregular features and topography of the mallard plantar was constructed.

In order to verify the sand fixation and flow-limiting characteristics of the morphological features, the mesoscopic behavior of mallard plantar particles was observed by discrete element simulation intrusion tests. The results showed that in the process of mallard foot intrusion in loose substrates, the disturbance of the infrafoot particles was mainly concentrated in the subfoot area, and the disturbance of the toe edge and the particles far away from the foot-end was small. The lateral webbed surface and the medial webbed surface make the foot particles move mainly downward to avoid diffusion to the periphery, which increases the density and bearing capacity of the plantar medium and can also reduce the foot subsidence, providing more stable support for the mallard to stand and move on the loose medium.

Finally, by extracting the morphological characteristics of the plantar of the mallard and applying the characteristics of the middle concave and edge bulge to the wheel grousers of paddy field wheels, two kinds of WBWGs were designed. Through the discrete element simulation test, the process of wheel grouser soil penetration is simulated, and it is found that compared with the traditional wheel grousers, the WBWG-C has better sand fixation, sand accumulation, and traction performance, which can reduce the damage to the soil layer when touching the soil and greatly improve the passing performance and anti-subsidence ability of agricultural equipment.

## Figures and Tables

**Table 1:** Lateral webbed surface fitting results.

Parameter	*xy*	*x* ^2^ *y*	*xy* ^2^	*x* ^2^ *y* ^2^
Sum of squares of residuals (SSE)	401,610	115,310	376,440	21,471
Mean square deviation (RMSE)	2.0103	1.0772	1.9463	0.4648
Coefficient of determination (R^2^)	0.7309	0.9227	0.7478	0.9856

Note: *x^n^y^m^* means that in this fitting equation, the exponent of *x* is maximum *n*, and the exponent of *y* is maximum *m*.

**Table 2:** Medial webbed surface fitting results.

Parameter	*xy*	*x* ^2^ *y*	*xy* ^2^	*x* ^2^ *y* ^2^
Sum of squares of residuals (SSE)	65,957	38,158	56,770	30,640
Mean square deviation (RMSE)	0.8669	0.6594	0.8043	0.5909
Coefficient of determination (R^2^)	0.9046	0.9448	0.9179	0.9557

Note: *x^n^y^m^* means that in this fitting equation, the exponent of *x* is maximum *n*, and the exponent of *y* is maximum *m*.

## Data Availability

The data presented in this study are available on request from the corresponding author. The data are not publicly available due to our laboratory privacy data protection.
